# Motor Training After Stroke: A Novel Approach for Driving Rehabilitation

**DOI:** 10.3389/fneur.2022.752880

**Published:** 2022-05-23

**Authors:** Neha Lodha, Prakruti Patel, Agostina Casamento-Moran, Evangelos A. Christou

**Affiliations:** ^1^Department of Health and Exercise Science, Colorado State University, Fort Collins, CO, United States; ^2^Department of Applied Physiology and Kinesiology, University of Florida, Gainesville, FL, United States

**Keywords:** stroke, driving rehabilitation, braking, processing, speed, movement, cognition, motor intervention

## Abstract

**Background:**

A key component of safe driving is a well-timed braking performance. Stroke-related decline in motor and cognitive processes slows braking response and puts individuals with stroke at a higher risk for car crashes. Although the impact of cognitive training on driving has been extensively investigated, the influence of motor interventions and their effectiveness in enhancing specific driving-related skills after stroke remains less understood. We compare the effectiveness of two motor interventions (force-control vs. strength training) to facilitate braking, an essential skill for safe driving.

**Methods:**

Twenty-two stroke survivors were randomized to force-control training or strength training. Before and after training, participants performed a braking task during car-following in a driving simulator. We quantified the cognitive and motor components of the braking task with cognitive processing time and movement execution time.

**Results:**

The cognitive processing time did not change for either training group. In contrast, the movement execution became significantly faster (14%) following force-control training but not strength training. In addition, task-specific effects of training were found in each group. The force-control group showed improved accuracy and steadiness of ankle movements, whereas the strength training group showed increased dorsiflexion strength following training.

**Conclusion:**

Motor intervention that trains ankle force control in stroke survivors improves the speed of movement execution during braking. Driving rehabilitation after stroke might benefit from incorporating force-control training to enhance the movement speed for a well-timed braking response.

## Introduction

Functional independence is a critical concern for maintaining quality of life after stroke (1). The ability to drive a car safely is at the heart of this concern. A large number of stroke survivors (33–66%) return to driving even in the absence of driving assessment and rehabilitation that retrains the skills necessary for safe driving (2–4). Identifying training interventions that facilitate driving-related skills is important for enabling return to driving safely and promoting functional autonomy after stroke.

Driving requires integration of motor and cognitive processes, both of which may be affected after a stroke (5). Emerging evidence suggests adverse implications of motor deficits after stroke on specific skills related to driving. For example, post-stroke impairments in upper limb force control contribute to steering dysfunction (6). Likewise, stroke-related impairments in lower limb motor accuracy are linked to slower braking response (7). Unsurprisingly, therefore, the severity of motor impairments after stroke predicts an individual's likelihood of self-regulating driving after stroke and returning to driving 6 months post-stroke (2, 4, 8). Although the impact of cognitive training on driving has been extensively investigated (9, 10), the influence of motor interventions and their effectiveness in enhancing specific driving-related skills after stroke remains less understood. Therefore, in this study we compare the effectiveness of two motor interventions (force-control vs. strength training) to facilitate braking, a driving-related skill that is essential for safe driving.

Well-timed braking response requires cognitive processing to recognize changes in the driving environment and motor capabilities for fast and accurate pedal manipulations. Accordingly, decline in cognitive speed of processing after stroke has been shown to delay the braking times (7). Although there is evidence that reduced muscle strength compromises braking speed in older adults (11, 12), our recent findings suggest that braking performance in stroke survivors relates to reduced motor control but not to muscle strength (7, 13). Specifically, we found that accuracy of ankle movements in a visuomotor force tracking task predicted the braking reaction time in stroke. Yet, ankle dorsiflexion and plantarflexion strength were uncorrelated to braking reaction in a simulated driving environment.

Here, thus, we examine the effectiveness of force-control and strength training motor interventions to facilitate braking performance in chronic stroke survivors. Braking performance was quantified using the cognitive processing speed and movement execution speed of the braking task conducted in a simulated driving environment. We hypothesized that both motor interventions will improve movement execution speed of braking, however, the magnitude of improvement will be greater in force-control training. This hypothesis was based on our previous work showing that ankle motor control, not ankle strength is linked to braking performance in stroke survivors (13). The current study is important because it will facilitate the development of motor interventions that could enhance braking performance and promote safe driving mobility in individuals with stroke.

## Methods

### Participants

Twenty-two chronic stroke survivors qualified to participate in the study. The inclusion criteria were: (1) diagnosis of stroke more than 6 months before study enrollment; (2) presence of a minimum of 5° of ankle plantarflexion and 15° of dorsiflexion range of motion; (3) ability to grasp a steering wheel; (4) ability to follow a three-step command; and (5) not involved in any other physical rehabilitation. Individuals were excluded in the presence of (1) uncorrected vision and hearing loss; (2) self-reported visual neglect; (3) diagnoses of sensory or global aphasia; (4) pain and musculoskeletal or any other neurological disorder. All participants read and signed an inform consent approved by the Institutional Review Board of University of Florida before participating in the study.

### Study Design

**Figure 1:**
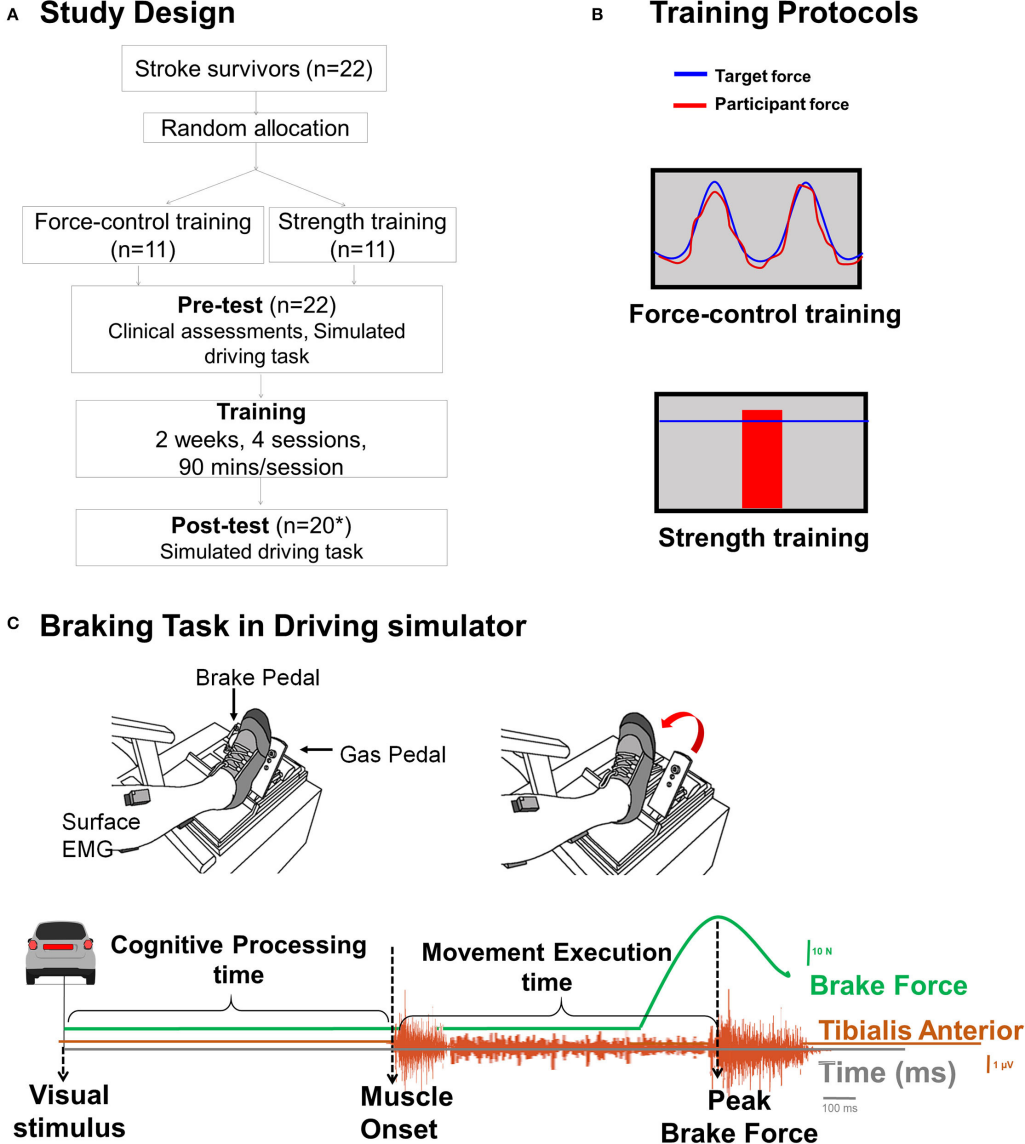
**(A)** Study design. We randomly allocated 22 individuals with stroke to force-control or strength training. *One individual in each training group was lost to follow-up due to inability to return for post-test driving assessment. **(B)** Training description: The *force-control training* group performed a visuomotor force tracking task that involved matching participant's ankle force to a sinusoidal force trajectory (top). Blue sinusoidal line represented the target trajectory with amplitude of 10% maximum voluntary contraction force (MVC) (range 5–25% MVC) and the red line shows the participant's performance. The difficulty of force-control training progressed by decreasing the target frequency from 0.2, 0.1, 0.05, to 0.1 Hz + 0.2 Hz over 4 sessions. The *strength training* group performed rapid muscle contractions to reach a target force (bottom). The target force was displayed with blue horizontal line that represented a pre-determined percentage of their MVC force and participant's performance was displayed with a vertical red bar. The difficulty of the strength training was progressed by increasing the target force from 65, 70, 75 to 80% of MVC force over 4 sessions. Both the groups performed the trainings with the driving leg in both contraction types (dorsiflexion and plantarflexion). **(C)** Time series of a single representative trial of braking task. The cognitive processing time was measured as the time between onset of the visual stimulus (brake lights of the lead car) and activation of tibialis anterior muscle. The movement execution time was measured as the time between the activation of tibialis anterior muscle activation to the peak brake force.

Twenty-two stroke survivors were randomly assigned to either force-control training or strength training group (Figure 1A). One participant in each group failed to return at post-test. Each group received 2 weeks of motor training in 4 sessions with increasing intensity. The training session lasted for 90 min. Before (pre-test) and after (post-test) the training, we evaluated the participants on a braking task that was performed in driving simulator to measure their cognitive processing time and movement execution time. At pre-test, we also performed clinical assessments including the lower extremity subsection of Fugl-Meyer Motor Assessment and Montreal Cognitive Assessment.

### Training Description

Figure 1B shows a schematic of the force-control and strength training protocols. The force-control group practiced a visuomotor task that involved tracking a sinusoid. The strength training group practiced fast motor contractions at a percent of participants' maximal voluntary contraction (MVC) force. Both training protocols involved unilateral, isometric contraction involving plantarflexion and dorsiflexion of ankle used for driving, while avoiding any extraneous movements at knees, hip, and trunk. During the training session, participants sat in an upright chair in front of a 32-inch monitor placed about 1.5 m away. The leg was positioned with hips and knees at ~90 degrees and ankles in a neutral position. The foot rested on a custom-built foot device and was secured to the device with straps. The plantarflexion and dorsiflexion MVCs were assessed at the beginning of each training session to determine the target forces for force-control and strength training. Additional details of the training have been reported in our previous publication (14).

### Force-Control Training

Force-control training required gradual increase and decrease in ankle forces to perform a visuomotor sinusoidal tracking task. Within each session, participants performed five sets of six trials for each contraction (plantarflexion and dorsiflexion). The order of the contraction type was randomized across participants. Each training set lasted 5 minutes. The training intensity was progressed on successive sessions by reducing the frequency of the target sinusoid.

### Strength Training

Strength training involved practicing fast ankle muscle contractions to produce a target force. Within each session, participants performed six sets of 15 repetitions for each contraction. The order of the contraction type was randomized across participants. Each training set lasted 5 min. The training intensity was progressed on successive sessions by increasing the magnitude of target force from 65 to 80% MVC.

### Pre- and Post-tests

To examine the effect of the motor training on braking performance, we measured cognitive processing time and movement execution time on the braking task during a simulated driving task before (pre-test) and after (post-test) both motor training protocols. To examine task-specific effects of force-control and strength training, we measured motor accuracy, motor steadiness, and ankle strength. The study was single blinded such that the participant's training group assignment was unknown to the examiner who conducted pre and post-test.

### Simulated Driving Task

We used a custom-built driving simulator to evaluate braking performance. Participants sat comfortably in the car seat of a professional driving simulator with customized gas and brake pedals (15). A 32-inch monitor in front of participants (Sync Master 320MP-2, Samsung Electronics America, Resolution: 1,920 × 1,080, Refresh Rate: 60p Hz) displayed the driving scenario. The driving task involved following a lead car by pressing and releasing the gas pedal. At random times, the brake lights of the lead car turned red. Participants responded to this visual stimulus by pressing the brake pedal as fast as possible and applying a controlled brake force to slow the car. After three practice trials, participants performed 10 test trials. Each trial lasted 20 s with a rest duration of 60 s between the trials. All individuals except two participants performed the driving task with their right leg (Table 1).

**Table 1:** Demographics of the participants in each training group (mean ± SD).

	**Force-control training** **(*N* = 10)**	**Strength training** **(*N* = 10)**
Age (years)	64.99 ± 10.11	65.95 ± 15.25
Sex (females), *N*	3	5
Hemiparetic side (right), *N*	7	9
Time since stroke (years)	6.55 ± 4.84	5.44 ± 5.94
**Lesion location**		
Cortical	8	5
Subcortical	2	2
Unknown	0	3
MoCA	24.50 ± 5.08	24.10 ± 3.75
FMA-LE	21.40 ± 9.66	25.90 ± 4.77
Driving leg (Paretic, *N*)	6	7

*MoCA, Montreal cognitive assessment (maximum score 30); FMA-LE, Fugl-Meyer motor assessment for lower extremity (maximum score 34); All scores are mean ± standard deviation*.

### Data Acquisition and Analysis

The gas pedal position was measured using CSR Elite Pedals (Fanatec, Endor AG, Germany). The gas pedal position data were sampled at 100 Hz and high-pass filtered with a cut off frequency of 3 Hz. The brake pedal force was measured using a force transducer (Model LAU200, 100 lb. capacity, FUTEK Advanced Sensor Technology, Irvine, CA) embedded on the brake pedal. The brake force data were sampled at 1,000 Hz and high pass filtered with a cut off frequency of 0.03 Hz. The tibialis anterior muscle activity of the driving leg was measured with wireless surface electromyography (EMG) electrode (Delsys Trigno, Delsys, Boston, MA). The EMG signals were sampled at 1,000 Hz with an NI-DAQ card (Model USB6210, National Instruments, Austin, TX, USA). The EMG data were band pass filtered at 20–450 Hz, amplified with a gain of 1000, and stored on a computer. The EMG signal for each trial was rectified and smoothed using fourth-order Butterworth filter with a cut off frequency of 6 Hz. The filtered EMG signal was used to identify the muscle onset. All the data were analyzed offline using a custom-written program in Matlab (Math Works Inc, Natick, MA, USA).

### Braking Outcomes

We assessed the braking performance on the driving task with cognitive processing time and movement execution time (Figure 1C). We quantified the cognitive processing time as the time from the onset of the visual stimulus (brake lights of the lead car) to the activation of tibilalis anterior. We quantified the movement execution time as the time from the activation of tibialis anterior to the application of peak brake force.

### Measurement of Training Effects on Ankle Movement Control

The ankle movement control involved tracking a sinusoidal target trajectory with isolated, ankle plantarflexion-dorsiflexion movements of the driving leg. The participant position was similar to training sessions except that the foot was secured to the custom-built foot device with straps such that the axis of rotation of the ankle aligned with the axis of rotation of the device, ensuring simultaneous movement of the foot and the device. *Task*: We measured ankle movement control of the driving leg during a visuomotor ankle movement tracking task. The target ankle movement ranged from 15 degrees of dorsiflexion to 5 degrees of plantarflexion. The target trajectory of 0.3 Hz was displayed with a red line on the computer screen in front of the participants. Participants were asked to perform rhythmic ankle dorsiflexion and plantarflexion movements to match the target trajectory as accurately as possible. A real-time feedback of participant's ankle movement was displayed with a blue line. Each trial lasted for 35 s. Three familiarization trials preceded the test trials. Participants performed five consecutive test trials involving ankle dorsiflexion and plantarflexion. A rest period of 30 s was provided between test trials to minimize fatigue.

### Ankle Position Analyses

The ankle position was measured with a low-friction potentiometer (SP22G-5K, Mouser Electronics, Mansfield, TX, USA) with a sampling rate of 1,000 Hz (NI-DAQ card, Model USB6210, National Instruments, Austin, TX, USA), connected to the axis of rotation of the foot device. A custom written algorithm in Matlab controlled the visual presentation of each trial and computed the outcome variables.

### Ankle Motor Accuracy and Steadiness

We quantified accuracy and steadiness of ankle movement on visuomotor tracking task by averaging across five test trials. The initial 10 s and the last 5 s of data from each trial were eliminated to allow for adjustment to the task and early cessation of the performance in anticipation of trial termination. The motor accuracy was measured using root mean squared error (RMSE) that quantified the distance between the target and participant's ankle position trajectories. The motor steadiness was measured as the standard deviation (SD) of participant's ankle position within each trial. For this, we first band-stop filtered the position signal between 0.2 and 0.4 Hz to remove the task-related frequency of 0.3 Hz sinusoidal target. Then, we quantified movement steadiness with the SD of the detrended position signal.

### Measurement of Training Effects on Ankle Strength

We examined the ankle plantarflexion and dorsiflexion strength by measuring the isometric MVC force of the driving leg. Participants sat in an upright chair with hip and knees at ~ 90 degrees and ankle in a neutral position. The maximum voluntary force was measured using a force transducer (Model 41BN, Honeywell, Morristown, NJ, USA) located parallel to the force direction on a customized foot device. *Task*: Participants were instructed to exert maximum force at the ankle joint during plantarflexion or dorsiflexion for a period of 3 s while avoiding any extraneous movements at knee, hip and trunk. Three to five trials were performed for plantarflexion and dorsiflexion. The MVC task order for contraction type was randomized across participants. The strength was quantified as the maximum force generated across three-five trials.

### Statistical Analysis

To examine the influence of motor interventions on braking outcomes, we used a 2 Training Group × 2 Time, mixed model analysis of variance (ANOVA) on (1) cognitive processing time, (2) movement execution time. Here, Training Group (force-control and strength training) was the between-subject factor and Time (pre-test and post-test) was the within-subject factor. To determine the task-specific effects of training, we used paired *t*-test on pre-test and post-test scores on (1) RMSE of ankle movement and, (2) SD of ankle movement for the force-control training group, as well as (3) MVC of plantarflexion and dorsiflexion for the strength training group. All analyses were performed using SPSS (SPSS Inc. version 24.0). The significance level was set at *p* <0.05 and the effect sizes are reported for significant results.

## Results

### Participant Characteristics

Table 1 shows the clinical characteristics of participants in the motor-control and strength training groups. The two groups were not significantly different in age (*t*_|18|_ = −0.16, *p* = 0.87), time since stroke (*t*_|18|_ = 0.45, *p* = 0.65), FMA score (*t*_|18|_ = −1.32, *p* = 0.20), and MoCA score (*t*_|18|_ = 0.20, *p* = 0.84).

### Cognitive Processing Time

**Figure 2:**
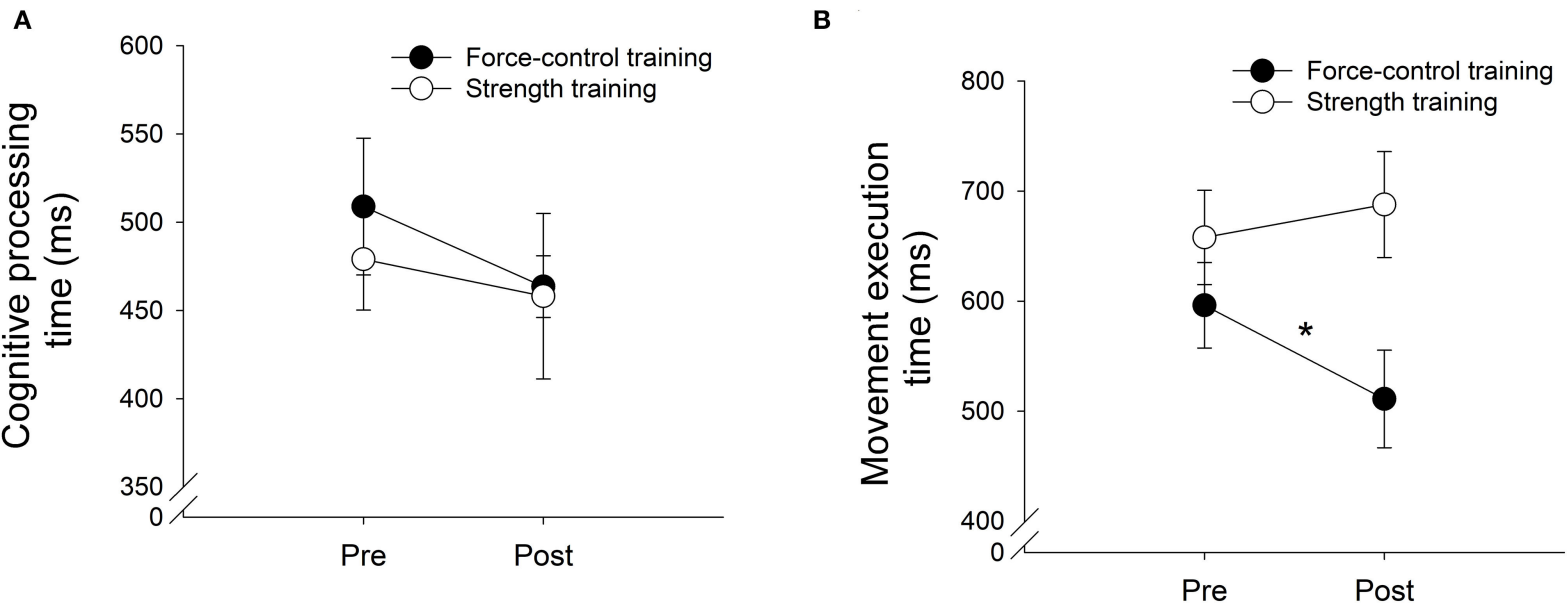
The cognitive processing time **(A)** and movement execution time **(B)** after force-control training (closed circle) and strength training (open circle). The cognitive processing time did not decrease significantly after for both motor interventions **(A)**. The movement execution time significantly reduced after force-control training but not after strength training **(B)**.

Figure 2A demonstrates the effects of both training protocols on cognitive processing time. We found no main effect of Training Group, (*F*_1, 18_ = 0.21, *p* = 0.65) or Time (*F*_1, 18_ = 1.18, *p* = 0.29). Further, there was no significant Training Group x Time interaction for cognitive processing time (*F*_1, 18_ = 0.16, *p* = 0.69, Figure 2A; Table 2).

**Table 2:** Two (training group) × two (time), mixed model analysis of variance on cognitive processing time and movement execution time.

**Dependent variable**	**Source**	**Sum of squares**	**df**	**Mean square**	**F**	**Sig**.	**Partial Eta squared**
**Cognitive processing time (ms)**							
	Time	10,995.74	1	10,995.74	1.183	0.291	0.062
	Training group	3,110.18	1	3,110.18	0.209	0.653	0.011
	Time * Training group	1,492.36	1	1,492.36	0.161	0.693	0.009
	Error within-subjects	167,344.00	18	9,296.88			
	Error between-subjects	267,597.12	18	14,866.50			
**Movement execution time (ms)**							
	Time	7,618.57	1	7,618.57	1.150	0.298	0.060
	Training group	142,032.32	1	142,032.32	4.511	0.05	0.200
	Time * Training group	33,068.76	1	33068.76	4.992	0.038	0.217
	Error within-subjects	119,244.01	18	6,624.66			
	Error between-subjects	566,743.51	18	31,485.75			

### Movement Execution Time

Figure 2B demonstrates the effects of training on movement execution time. We found a significant main effect of Training Group, (*F*_1, 18_ = 4.51, *p* = 0.04; η^2^ = 0.20) on movement execution time. Importantly, we found a significant Training Group x Time (*F*_1, 18_ = 4.99, *p* = 0.03, η^2^ = 0.22, Figure 2B; Table 2) interaction. The descriptive statistics show that while the force-control training group reduced movement execution time at post-test compared with pre-test (Pre-test *M* = 596.22, SD = 122.85 ms; Post-test *M* = 511.11, *SD* = 135.45 ms), the strength-training group increased movement execution time after training (Pre-test *M* = 657.89, SD = 135.45 ms; Post-test *M* = 687.79, *SD* = 152.18 ms). Specifically, we found a 14% reduction in movement execution time for the braking movement following force-control training vs. 6.04% increase in movement execution time after strength training.

A secondary analysis showed that the two training groups did not differ on the peak brake force values at post-test (*t*_|18|_ = −1.45, *p* = 0.16) or pre-test (*t*_|18|_ = −0.004, *p* = 0.99) confirming that the changes in movement execution speed obtained following training were independent of the group differences in the peak force reached by each group. Finally, a Pearson's bivariate correlational analysis was conducted to test if improvements in movement execution speed were linked to the changes in motor control following training. We found no correlation between changes in ankle movement control in force control training group and movement execution speed (*r*_*accuracy*_ = 0.03, *p* = 0.93; *r*_*steadiness*_ = −0.56, *p* = 0.11).

### Task-Specific Effects of Training Protocols

#### Force-Control Training

**Figure 3:**
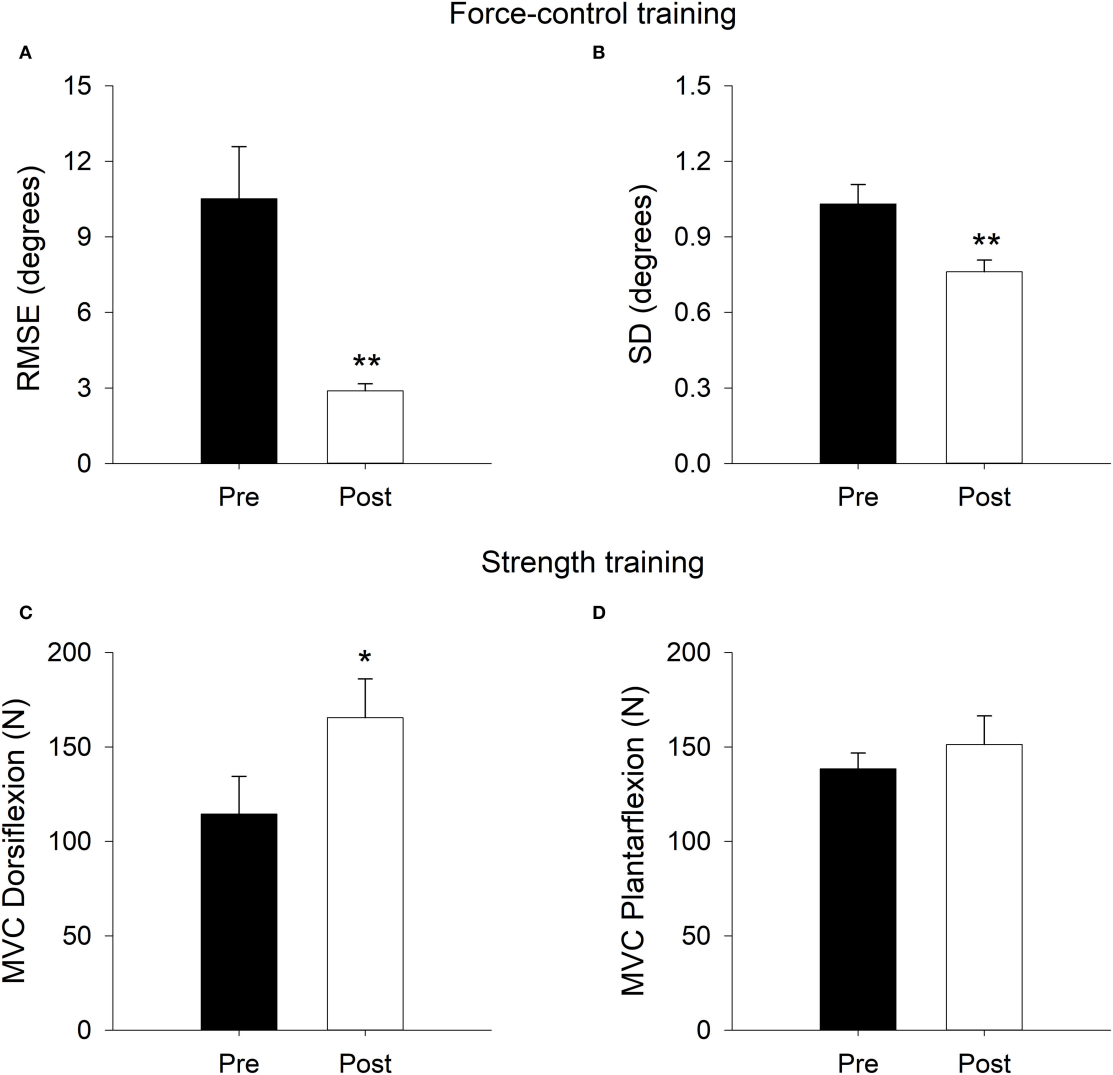
Task-specific training effects on ankle movement error (Root mean square error, RMSE; **A**) and ankle movement steadiness (standard deviation, SD; **B**) in the force-control training group. Task-specific training effects on dorsiflexion strength (maximum voluntary contraction; MVC; **C**) and plantarflexion strength **(D)** in the strength training group. The ankle movement accuracy and steadiness improved after force-control training. The ankle dorsiflexion strength improved after strength training. **p < 0.01; *p < 0.05.

Figures 3A,B demonstrates the results for motor accuracy and steadiness in force-control training group. The force-control training group showed improved accuracy (*t*_|8|_ = 3.80, *p* = 0.005, *d* = 1.26) and steadiness (*t*_|8|_ = 4.85, *p* = 0.001, *d* = 1.62) of ankle movements following training.

#### Strength Training

Figures 3C,D demonstrates the results for ankle plantarflexion and dorsiflexion strength in strength training group. The strength training group showed improved dorsiflexion strength (*t*_|9|_ = −2.20, *p* = 0.027), although, plantarflexion strength (*t*_|9|_ = −0.76, *p* = 0.23) did not show statistically significant change after training.

## Discussion

The current study examined the effectiveness of motor interventions on braking performance in chronic stroke survivors. We compared the effect of force-control training and strength training on cognitive processing and motor execution time during braking in simulated driving environment. The movement execution time for braking response showed a 14% reduction after force-control training. The cognitive processing time for braking response did not change after either force-control or strength training. We found training specific improvements in ankle motor accuracy and steadiness after force-control training and increased dorsiflexion strength following strength training. Taken together, our findings demonstrate the effectiveness of force-control training in facilitating braking performance after stroke through improvement in movement execution speed.

The current study is the first to examine the effectiveness of motor interventions on driving related skills after stroke. To-date, driving rehabilitation studies in stroke survivors that emphasize improvements in underlying driving skills have primarily focused on cognitive training for enhancing the speed of processing. Cognitive training has shown promising results in improving choice reaction time task in simulated driving and enhancing the likelihood for success on on-road driving evaluation tests (10, 16). However, no study to date has investigated the effect of motor training on driving related outcomes. Unequivocally, fast, and accurate limb movements are essential for successful manipulation of car-controls (including brake and gas pedals) to drive safely (6, 13, 17). Our study pioneers the investigation of determining the effectiveness of motor interventions for improving movement speed during braking performance in individuals with stroke. We specifically focused on braking because impaired braking constitutes one of the highest risk factors for car crashes (18–20). Therefore, the current study represents a critical, first-step toward understanding the potential of targeted motor interventions for improving ability to execute fast limb movements for a well-timed braking response in stroke survivors.

A noteworthy feature of our study is that we compared two distinct forms of motor interventions to improve the cognitive processing and movement execution speeds involved in braking performance. We found a 14% reduction in movement execution time after force-control training. The movement execution time remained unchanged after strength training. Two key factors explain these findings. *First*, during force-control training participants practiced modulating small amounts of ankle forces that are essential for applying a controlled and efficient brake force. In contrast, during the strength training, participants produced quick and large ankle forces that did not require fine control. Potentially, force-control training improved the driver's ability to accurately modulate the timing and amplitude of ankle forces, which in turn contributed to faster movement execution time while braking. In line with our findings, a previous study in stroke survivors showed that a motor training where participants practiced controlled wrist movements with the paretic hand improved motor speed on symbol digits modalities test (21). In another study, visuomotor training for finger flexion-extension reduced movement error and improved the time required to respond to sudden changes in the target movement trajectory (22). Our findings extend these previous findings by showing the potential utility of ankle force-control training for improving driving-related motor skills such as movement speed of braking. *Second*, force-control training required ankle force modulation in response to real-time visual feedback of the target and participant's performance. Thus, participants learned to respond quickly and accurately to changing visual information with accurate modulation of the exerted ankle forces. Such ability to integrate and respond to visual information with the appropriate motor action is an essential component of braking performance in a simulated driving environment. Thus, it is possible that force-control training improves the way participants utilize and respond to visuomotor information with controlled ankle forces required for successful brake pedal manipulation.

The task-specific effects of training protocols confirmed that force-control training improved ankle movement control. Specifically, we found a 22.79% greater accuracy and 23.92% greater steadiness of ankle movements following force-control training. Interestingly, these changes in movement control resulted from motor intervention that trained force-control. Seminal work on neurophysiology of movement suggests that force-control training is associated with adaptations in motor-unit firing and recruitment profiles that may result in improved movement speed (23). Another explanation for training-related shortening of movement time during braking performance (without any changes in the cognitive processing time) could be that force-control training altered the distribution of activation among agonist and antagonist muscles resulting in improved muscle coordination between dorsiflexors and plantarflexors, and a consequent increase in movement speed. While mechanisms underlying force-control training induced improvement in movement speed are a subject of further investigation, our findings certainly demonstrate the effectiveness of force-control training in facilitating braking performance after stroke through improvement in movement execution time.

It is important to note that the braking task in the current study required controlled braking rather than hard braking. Specifically, the driver was required to apply sufficient ankle force to slow the car's speed in a fast and controlled manner rather than slamming the brakes with maximal forces to completely stop the car. Thus, force-control training improved the speed of the movement execution during a controlled braking task. Whether these results hold true when testing participants under hard braking situations (e.g., when a pedestrian or a dog runs in front of the driver's car) is unclear. Further, our findings confirmed that there was no significant difference in peak brake forces applied at post-test by both training groups. These results strengthen our findings by confirming that the faster movement speed in force-control training group were not simply obtained because of lower peak brake forces but rather due to faster rate of brake force application. Overall, these findings suggest that a motor intervention that trains accurate ankle force modulation has the potential to enhance the movement execution speed of a controlled braking response.

### Considerations and Conclusions

This proof-of-concept, preliminary study in chronic stroke survivors provides the first line of evidence regarding the impact of motor intervention on producing small but statistically significant changes in the speed of movement execution during braking in driving simulator. The current study included only 22 chronic stroke participants, based on the sample size recommendations appropriate for pilot studies (24). Our stroke cohorts had mild to severe lower limb motor impairment as measured by Fugl-Meyer assessment. Future randomized controlled trials will be needed to confirm these findings with bigger datasets and generalize the findings to a larger stroke population. Most of the stroke survivors in our study demonstrated mild to moderate motor impairments in lower extremity. Further investigation is required to understand the effectiveness of these motor interventions on braking outcomes in individuals with more severe motor impairments that continue to drive. Interestingly, neither motor interventions were effective in improving the speed of cognitive processing required for braking performance. Thus, cognitive training seems to be essential to improve the speed of cognitive processing required for driving (9, 10). Although driving in simulator provides a safe and controlled environment to test driver's behavior to hazardous condition, future studies should investigate whether improvements in movement speed during braking in a simulated environment would translate to on-road braking. Finally, our study involved a brief training period that might not be sufficient for inducing a longer-term improvements in strength and motor control. Studies investigating dose-response effects of strength and force-control trainings for braking performance are warranted.

In summary, the current study provides empirical evidence that motor interventions that train ankle force modulation in stroke survivors may provide a promising approach for improving the speed of movement execution for fast braking response, an essential skill for safe driving after stroke. Driving rehabilitation after stroke should consider incorporating force-control training to enhance the movement speed for a well-timed braking response.

## Data Availability Statement

The raw data supporting the conclusions of this article will be made available by the authors, without undue reservation.

## Ethics Statement

The studies involving human participants were reviewed and approved by Institutional Review Board of University of Florida. The patients/participants provided their written informed consent to participate in this study.

## Author Contributions

NL and EC conceived and designed the experiments. NL and AC-M conducted data collection. PP conducted data analysis. PP and NL did data interpretation and drafted the manuscript. PP, AC-M, EC, and NL revised and approved the manuscript for publication. All authors contributed to the article and approved the submitted version.

## Funding

The American Heart Association (Scientist Development Award 14SDG20450151 to NL) and National Institutes of Health (R21NS096258 to NL and EC) provided the funding support for this work.

## Conflict of Interest

The authors declare that the research was conducted in the absence of any commercial or financial relationships that could be construed as a potential conflict of interest.

## Publisher's Note

All claims expressed in this article are solely those of the authors and do not necessarily represent those of their affiliated organizations, or those of the publisher, the editors and the reviewers. Any product that may be evaluated in this article, or claim that may be made by its manufacturer, is not guaranteed or endorsed by the publisher.
